# Risk Perception, Perceived Government Coping Validity, and Individual Response in the Early Stage of the COVID-19 Pandemic in China

**DOI:** 10.3390/ijerph20031982

**Published:** 2023-01-21

**Authors:** Tao Xu, Mengyuan Shao, Ruiquan Liu, Xiaoqin Wu, Kai Zheng

**Affiliations:** 1 School of International Culture and Social Development, Zhejiang Normal University, Jinhua 321000, China; 2 Department of Economics and International Trade, School of Economics, Management & Law, Hubei Normal University, Huangshi 435002, China

**Keywords:** risk perception, perceived government coping validity, individual response, COVID-19 pandemic

## Abstract

As a major crisis event, the COVID-19 pandemic has affected the global economy, threatened the lives of the public, and caused varying degrees of impact on the public. Previous studies have shown that risk perception and government response had different impacts on the public, but they revealed more about the independent impact of risk perception and government response on the public. This study will comprehensively consider the impacts of these two factors on the behavior of the public in the early stage of the epidemic. We analyzed data from an online survey in the early days of the COVID-19 pandemic in China and categorized individual behaviors into three dimensions: entertainment and travel, work, and the stockpile of supplies. In addition, we defined the risk perception variables by two dimensions: knowledge of the epidemic itself and knowledge of the consequences of the epidemic. At the same time, we used an exploratory factor analysis to construct the variable of perceived government coping validity and then adopted the ordinal logit model for analysis. The results showed that in terms of entertainment and travel, people would not be affected even if they fully understood the epidemic itself; once they were aware of the negative social consequences of the epidemic, people would suspend entertainment and travel to prevent the spread of the virus. As for work or employment, people would not stop working or employment even if they realized the infectivity and harmfulness of the disease and its social consequences. Furthermore, fear of COVID-19 and the perception of uncontrolled COVID-19 significantly positively affected people’s material stockpiling behavior. These results indicate that different risk perceptions had different effects on individual responses, and individual behaviors reflected different coping logics. In addition, the government’s effective response to the epidemic would significantly reduce the negative impacts of the epidemic on the three dimensions of people’s responses. These conclusions have certain policy implications for preventing and responding to outbreaks in other countries.

## 1. Introduction

In late January 2020, coronavirus disease 2019 (COVID-19) first broke out in China and then swept worldwide. As a global public crisis, it has posed a threat to human life and uncertainty and a massive obstacle to developing the global economy and culture [1]. Since the outbreak of COVID-19, countries worldwide have suffered tremendously economically, culturally, and socially and faced threatened human physical and mental health [2]. The threat of the virus led governments worldwide to close borders, quarantine and keep social distance, and reduce travel outside the home to combat the spread of the outbreak [3,4]. Research addressing this issue has involved multiple research fields and multiple research perspectives: some studies explored coping strategies from organizational and managerial perspectives [5,6]; some studies explored the formation of and changes in people’s attitudes toward risky activities [7,8]; and some scholars emphasized the social construction of risk interpretation and its relationship to knowledge acquisition, social interests, and cultural values from a theoretical perspective [7,8,9]. These studies helped improve our understanding of individual risk perceptions and risk-coping responses. However, necessary attention has been lacking regarding the extent to which people’s daily lives and even entertainment activities were affected beyond risk response behaviors, and how people coped in these areas. In particular, it remains to be further explained how people’s determination and perception of risk and their evaluation of the validity of the government’s response influenced their daily work, life, and entertainment. This paper uses data from a survey conducted in China during the early stage of the COVID-19 pandemic to reveal their potential relationships.

### 1.1. Pandemic Prevention Actions during COVID-19

The outbreak of COVID-19 swept across the world, affecting people’s lives, work, and study. Governments, societies, and individuals worldwide took various measures to cope with it. In the early stages of the COVID-19 pandemic outbreak, governments of different countries around the world adopted policies such as the suspension of international flights, restriction of movement, social distancing, and quarantine to prevent the development of a widespread of infection of the COVID-19 in their countries [10]. For example, Wuhan, China, adopted a quarantine policy from 23 January to 8 April, 2020 [11]; Australia followed an influenza health management plan to adopt a policy of border management, self-isolation, and social distancing [12]; and Norway adopted economic policies to control the outbreak [13]. 

Actions at the social level to prevent the pandemic were equally important. Winslow expressed: “Public health is the science and art of preventing disease, prolonging life, promoting physical health, and improving effectiveness through organized community efforts…that requires the participation of social workers along with physicians, nurses, bacteriologists, epidemiologists, and other multicultural, highly competent individuals” [14]. Using the example of the pandemic in China, communities played a crucial role in pandemic prevention and control [15]. Zhejiang Province, China, had a record year of success during the early stage of the pandemic because the government first encouraged community organizations to participate in pandemic prevention, established a platform to update pandemic data, and established a long-term community responsibility mechanism [16]. The deployment of resources by the state to enrich the community and build a strong pandemic prevention network was an essential lesson for mainland China in controlling the spread of COVID-19 and restoring life and production within the early months of the pandemic.

After the outbreak, the government issued many individual behavioral recommendations for dealing with COVID-19. Most individuals followed these recommendations and took preventive measures such as going out less, wearing masks, and washing their hands more often [17]. Although different individuals almost always took appropriate countermeasures, there were some differences. A study of German university students showed that approximately 80% of students increased their preventive behaviors of wearing masks and washing hands during the COVID-19 pandemic; 51.8% and 38.2% were more careful about cleaning and using disinfectants, respectively; and a higher percentage of female students engaged in preventive behaviors compared to male students [18]. A behavioral risk factor surveillance report of 1000 respondents in Florida showed that most respondents reported their preventive behaviors during the outbreak, with 97% reducing or avoiding public activities with large crowds and 87.5% preferring to stay home [19]. One study showed that healthcare workers on the front lines of the fight against the COVID-19 pandemic were not immune to the psychological and mental health consequences of the COVID-19 pandemic. However, healthcare workers used problem-centered and emotionally focused coping behaviors to manage the stress of COVID-19, such as communicating with family members for emotional support and engaging in physical activities to relax from stress [20]. Another study showed significant differences in the health behaviors of many people before and after COVID-19, with those more negatively affected by the COVID-19 outbreak more often reducing their eating behaviors and participation in physical activity [21]. In addition, a scarcity theory-based study suggested that once the public was aware of the severity of the public health events, given the scarcity of necessities, they were likely to make irrational consumption behaviors such as hoarding large quantities of household goods [22]. The reason for this was probably that the emergence of the COVID-19 pandemic increased public stress and drove their irrational consumption behavior [23]. 

### 1.2. Risk Perception and Individual Response

Risk perception is an individual’s perception and awareness of objective risks in the outside world, emphasizing the influence of individual experience gained from intuitive judgments and subjective feelings on individual cognition. After the experience gained through perception was fed back to the individual, people inferred the feasibility of the experience and then took following action [24]. Many studies examined the relationship between individual responses and risk perception. These individual responses included coping, self-protection, and consumer behavior for emergencies.

As for coping response, several studies showed that the public’s risk perception could directly influence their coping response behavior in emergencies [25,26]. The higher the perceived risk was, the more people tended to take risk-reducing coping measures because the high risk put people in a negative emotional state, which naturally motivated them to take action to mitigate this state [27]. However, if the public had limited awareness of the risks and distrusted authoritative information, and in the absence of effective communication, the public would often act irrationally when faced with threats, such as spreading rumors and looting goods, resulting in risks being magnified [28]. In the context of public health emergencies, many social instability events originated from the differences in risk perceptions of different stakeholders and the coping responses that resulted from the differences. Based on the different levels of risk perception, the public tended to engage in coping responses such as searching for and disseminating risk information and evacuating risk areas to avoid risk and uncertainty [29]. 

For self-protective behavior, it was suggested that the perception of risk influenced self-protective behavior. Lower risk perception led to a lower willingness of the public to adopt self-protective behaviors; conversely, the higher the risk perception was, the stronger the willingness of the public to make self-protective behaviors was [30]. Current risk perception is considered to be an essential predictor of self-protective behavior. Some medical studies showed that the trajectory of infectious diseases is often closely related to the behavior of individuals, which in turn is related to risk perception [31]. During the COVID-19 pandemic, most people adopted self-protective behaviors, such as maintaining social distance, washing hands frequently, and disinfecting lights to avoid infection [32]. However, many people still did not take the appropriate protective measures, which undoubtedly increased their chances of contracting COVID-19 [33,34,35,36]. In addition to differences in gender, age, and personality, an important reason why people behaved differently was that they perceived the risk of the COVID-19 pandemic differently [37]. 

For consumption behavior, some studies showed that during the pandemic, members of the public with a higher risk perception tended to adopt consumption behaviors such as rush shopping. This was because they managed to reduce their risk of being infected due to self-protection mechanisms and their own needs [38]. The COVID-19 pandemic threatened global food safety and changed consumer purchasing and consumption behaviors. A study of the pandemic in Spain showed that consumers’ risk perception was an essential factor influencing consumers’ purchasing preferences [39]. A study of the Japanese population’s perception of the new coronavirus and willingness to purchase vaccines showed that when people rated the COVID-19 outbreak as a more severe disease, they perceived a higher probability of infection and were more willing to pay for the vaccine [40]. 

For work employment behavior, healthcare workers were at greater risk of infection as their occupation was directly exposed to the new coronavirus during the COVID-19 pandemic. Nearly 90% of healthcare workers believed that their work increased their risk of exposure to the new coronavirus and increased the probability of their family members being exposed to it, which led to more significant psychological stress among healthcare workers at work [41]. Some healthcare workers, recognizing such a high risk, chose to quit their jobs to reduce the likelihood of infection [42]. Moreover, a relevant study proved that the COVID-19 pandemic worsened the perceived economic environment, and many employees developed a strong tendency to leave their jobs [43], while some were even forced to give up their current jobs [44]. One study examined the factors influencing employee turnover behavior: perceived external employability and organizational growth. The study found that the severity of COVID-19 reduced employees’ awareness of external employability, reducing their possible turnover behavior. At the same time, the intensity of COVID-19 strengthened employees’ negative perceptions of organizational development and thus enhanced their turnover behavior [45].

### 1.3. Perceived Government Coping Validity and Individual Response

A public health emergency is a significant test of a government’s response capability. A government’s coping validity is the comprehensive ability of the government to use various resources and management tools to remedy and control the crisis after a public emergency and to cope with the public’s demands in a timely and effective manner [45,46]. In the context of the pandemic, this ability to respond was closely related to factors such as the government release of information about the outbreak and government trust. At the same time, the perceived government coping validity affected the behavioral performance of individuals during the pandemic.

The public was vulnerable during the outbreak, and their behavior was influenced by their perceptions and the external environment, resulting in various positive or negative responses. When a pandemic is threatening the public’s safety and security, the government must make policy decisions or issue policy information to guide the public to respond to the pandemic [47]. However, there were two scenarios: when the government released information or policies that met people’s expectations, people would support the government’s policies and respond positively; on the contrary, if the government released statements or policies that went against people’s wishes or perceptions, people would react negatively and lose trust in the government. The lack of trust made it difficult to stimulate supportive behavior in an uncertain situation [48]. This demonstrated that in a sudden public health event, government policies and information could directly influence the following action of the people. 

When a public crisis arises, if the government accurately and timely releases adequate information to remedy the trouble and cope with public demands promptly, the public would be more likely to take action to support government initiatives during the crisis event [49]. Moreover, it might even form a solid social organization mobilization capacity to a certain extent [50], and the public and the government could resolve the crisis together. On the contrary, if the government releases untimely information and policies, takes inappropriate measures, and fails to cope with the people’s demands effectively, it may lead to the rapid spread of the crisis and endanger people who may be helpless in the face of the crisis. 

One study further suggested that the government’s coping ability was directly related to people’s actions. The less familiar people were with the government’s measures and information to control the epidemic, the more nervous they were and the more likely they were to act irrationally [51]. A different study showed that the timing of government releases of policies and initiatives in response to the pandemic was associated with people adopting protective behaviors. It found that if the government took measures such as restricting actions within one week before the outbreak of the epidemic, people would be able to take timely self-protection actions to avoid infection and reduce the number of deaths [52]. Another study explored the relationship between Portugal’s emergency quarantine policy and public health behavior at the beginning of the epidemic. Specifically, the government issued epidemic prevention policies in a timely manner during the outbreak of COVID-19. As a result, nearly 80% of the population complied with the government’s epidemic prevention policies and adjusted indoor activities [53]. Thus, it was clear that the government could effectively adjust the public’s behavior and reduce the public’s infection rate if it issued the anti-pandemic policy promptly. 

As for government trust, Tummers argued that to better implement policies, the government should rely on people’s support and trust to guide and change their behavior [54]. In layperson’s terms, for people to trust the government, the government must have a solid ability to respond, especially to crises. The stronger the government’s ability to respond is, the more people would trust the government. A study on the credibility of the government showed that during the pandemic, the government should timely announce accurate information, tell the truth, respond to public needs, and establish an effective channel to communicate and feedback; otherwise, the credibility of the government would be weakened, and the implementation of the pandemic prevention policy would be greatly compromised [55]. Therefore, the government should achieve a timely and effective response to the pandemic, enhance its credibility, and gain the public’s trust so that the public can effectively cooperate and strictly comply with the government’s pandemic prevention initiatives. The government could thus control the spread of the pandemic and restore economic and social production on time. 

### 1.4. Analytical Framework and Research Hypothesis

We found that the government, society, and individuals were involved in the response to the pandemic in past studies [10,56,57,58,59]. The previous research has two significant weaknesses. First, much attention was paid to coping behaviors, such as self-protective behaviors, regarding the behavior of individuals, while relatively few studies were conducted on everyday behaviors related to work, life, recreation, and shopping brought about by the pandemic. Few studies included these behaviors in the same analytical framework. Second, although a relationship between risk perception and individual response was found, the perception of risk perception itself and the perception of risk consequences have not been distinguished by most studies.

**Figure 1:**
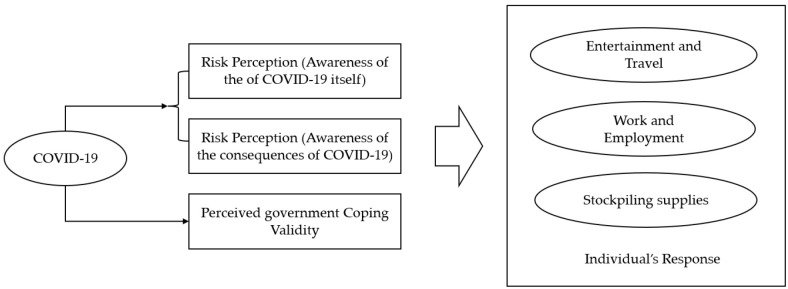
The conceptualized framework of risk perception, perceived government coping validity, and individual response.

Based on the above shortcomings, this paper formally constructed a new analytical framework (Figure 1). In this framework, we divided risk perception into the risk perception of COVID-19 itself and its consequences. At the same time, we categorized individual coping behaviors into three dimensions: entertainment and travel, stopping work or employment, and stockpiling of goods. This study investigates how different risk perceptions affected people’s daily entertainment and travel, work or employment, and stockpiling of supplies under the influence of the pandemic. We also examined how different judgments about the validity of the government’s response to the same risk perceptions affected people’s entertainment and travel, work or employment, and stockpiling of supplies. 

Past research showed that risk perception was closely related to individual behavior, and risk perception guided individuals’ actions to some extent [48,60,61]. People take different actions based on their judgment of the current risk, but many studies did not dimensionally delineate risk perceptions. According to the previous studies [62,63], at least two dimensions of risk perception were generated during the spread of the pandemic: one was risk judgment or perception of the virus itself based on previous similar public crises, while the other was the public’s risk perception based on the severe consequences of the current mutated virus. In this study, we categorized risk perception into the perception of the pandemic itself and its consequences. We argue that the effects of people’s actions after recognizing the characteristics of the epidemic and the consequences of the epidemic were different.

In terms of entertainment and travel, the COVID-19 pandemic caused people’s travel space to be constrained by objective conditions such as quarantine policies, traffic restrictions, and other measures. At the same time, people’s travel psychology also changed, the main reason for which lay in the risk perception of tourist travel, which was formed by the environmental pressure of entertainment and travel, which in turn lowered people’s willingness or motivation to travel [64]. However, this risk perception was usually formed by people from a consequence perspective, which was more guided by the fact that people started from a consequence perspective. They might have made trade-offs in their actions if the consequences caused more impact. The higher the level of people’s consequence-based risk perception is, the more cautious their behavior would be. People might behave differently if they start by perceiving an event itself. For example, if people know that there is a risk of drowning when going to the beach, but this hazard does not occur, people would still not give up going to the beach. The same is true for a pandemic. If people knew that there was a hazard and that it spread rapidly, but it did not become uncontrollable or cause death, people would still choose to travel or continue entertainment, even though this behavior increased the possibility of infection with COVID-19. Therefore, we made the following hypotheses:

**Hypothesis 1:**  *Different dimensions of risk perception had different effects on entertainment and travel. Awareness of the pandemic led to no suspension in entertainment and travel, and the understanding of the consequences significantly increased the probability of suspending their entertainment and travel*.

In terms of work or employment, from the perspective of people’s perceptions of COVID-19, although people realized that COVID-19 was infectious and harmful and that they might be infected, they still did not stop working [65] because work was a source of livelihood and a means of survival. However, when the pandemic was out of control, people might have made trade-offs with work, and even if they could earn income through work, protecting their lives might be more critical in the face of the potentially fatal outcome of COVID-19.

**Hypothesis 2:**  *Different dimensions of risk perception had different effects on work or employment; the perception of the pandemic did not make people stop working and employing, and the perception of the consequences of the pandemic increased the possibility of stopping working and employment*.

In terms of material supplies, in general, when influenza or common infectious diseases appeared, people did not increase their purchases of supplies because common epidemics were manageable and could usually be solved by purchasing medicines or going to hospitals [66,67]. When the COVID-19 pandemic first appeared, although people knew it was an unusual infectious disease, they had limited knowledge about it and did not hastily stockpile supplies. However, once people realized the extraordinary social consequences of the COVID-19 pandemic, they would flock to supermarkets and stores to grab supplies out of a need for safety and to maintain basic survival necessities in case of being quarantined and unable to go out and buy everyday items.

**Hypothesis 3:**  *Different dimensions of risk perception had different effects on the stockpiling of supplies. The perception of the pandemic did not significantly affect the stockpiling of supplies, while the perception of the consequences caused by the pandemic significantly affected people’s stockpiling of supplies*.

Government response validity was closely linked to people’s trust and behaviors. Different values may have led to different response policies. Countries believing the virus could not be eradicated never restricted people’s entertainment and travel during the pandemic but tried to coexist with the virus [68]. Some other countries were different; for example, during the past three years of the epidemic, China and some other countries adhered to the idea that only by strictly preventing the spread of the viruses could they protect people to the greatest extent [69]. They were strict in preventing virus spread and hoped to protect people through the zero-clearing policy. Based on the principle of strict control, very often, the government would restrict people’s travel and related entertainment activities to cut off the flow of people to prevent the pandemic’s spread [70,71]. Conversely, once the government could effectively cope with the pandemic and control the development trend, people could return on the right track in their lives and continue their entertainment, travel, and work at ease. This leads us to the following hypothesis:

**Hypothesis 4:**  *There was a significant adverse relationship between government response validity and suspension of entertainment and travel as well as stopping work or employment*.

During the pandemic, the government not only formulated various policies and took various measures to deal with the epidemic but also ensured the supply of all kinds of materials, especially medical protective items such as disinfectants and masks. If the government failed to ensure the provision of emergency supplies, it would probably directly lead to many people not being able to purchase protective supplies, which in turn would exacerbate the tension and cause wider hoarding of goods. If the government could effectively respond to the above situation, people could purchase items according to their daily needs without a widespread rush to buy them.

**Hypothesis 5:**  *There was a significant adverse effect relationship between governmental response validity and the stockpiling of supplies*.

## 2. Materials and Methods

### 2.1. Data

This survey was an online survey conducted in the early COVID-19 epidemic period in February 2020. The survey involved releasing online questionnaires through WeChat and QQ by using the snowball method to find more interviewees. A total of 1613 questionnaires were collected. The questionnaire data covered 30 provinces, municipalities, and autonomous regions in China. After deleting 76 missing surveys because more than one-third of the questions were not answered, we used 1537 responses. Although most of the initial questionnaire respondents were acquaintances, the questionnaire information collected by the snowball sampling method, compared with the current relevant demographic data, was almost consistent in most of the indicators except for some specific population variables; therefore, it is representative to some extent [58,72]. Moreover, the respondents filled in the questionnaire without any economic incentives, so the data collected are likely to be more realistic. Despite the limitations of online questionnaires, they still had practical significance given the situation of the COVID-19 outbreak.

### 2.2. Measures

#### 2.2.1. Dependent Variable

This study focused on the impact of the pandemic on individual responses. The questionnaire was designed to determine whether people would suspend entertainment and travel, stop working, and stock up on supplies, with the options of “strongly disagree”, “disagree”, “neutral”, “agree”, and “strongly agree”. The analyses were assigned values from 1 to 5 in that order.

#### 2.2.2. Independent Variables

##### Core Explanatory Variables

*Risk perception.* Slovic interpreted a risk event as a signal, and the nature of the signal itself and the conditions of the communication process affect the audience’s reception and interpretation of the event [63]. People often depend on intuition to recognize and judge risk events, and such intuition-based recognition and judgment were called risk perception [73]. The Chinese scholar Liu, on the other hand, divided risk perception into controllability, visibility, fearfulness, likelihood, and severity of risk [74]. This paper divided the public’s risk perception into the perception of COVID-19 itself and the perception of the consequences of COVID-19. First, the public perception of the COVID-19 pandemic outbreak included the fact that COVID-19 was highly contagious, that people feared COVID-19, and that COVID-19 could get out of control. The responses to these three variables included “strongly disagree”, “disagree”, “neutral”, “agree”, and “strongly agree”; we categorized these five levels by combining “strongly disagree” and “disagree” into “disagree” with a value of 0 and combining “neutral”, “agree”, and “strongly agree” into “agree” with a value of 1. Secondly, public awareness of the consequences of the pandemic was measured by a scale that included the degree of concern about COVID-19, the degree of personal effect, and the social effect. The reliability analysis showed that the scale had a value of α = 0.65, the factor analysis KMO value was 0.78, and the reliability and validity passed the test. Using principal component analysis, we performed factor rotation on the scale and extracted three factors. Concerning Xie and Slovic et al. [73,75], the three factors of “anxiousness, potential impact, and controllability” were extracted from people’s perceptions of the consequences of the pandemic by exploratory factor analysis (Supplementary Table S1).

*Perceived government coping validity.* Perceived government coping validity is the public’s subjective evaluation of the response measures taken by the government during the pandemic. In exploring the impact of perceived government coping validity on government credibility during the COVID-19 pandemic, Xue classified the perceived government coping validity into four aspects: proactiveness, responsiveness, transparency, and accuracy [8]. It was seen that there was a mutual inclusion of proactivity, responsiveness, transparency, and accuracy in Xue’s delineation. Based on this study, it was reasonable to delineate multiple dimensions to reflect the government’s response validity through the public’s subjective evaluation of its response measures during the pandemic. At the same time, factor analysis could avoid this problem by integrating the dimensions into one part to reflect the government’s response validity. In our survey, there was a group of items to ask citizens to judge the effectiveness of the government’s coping behavior (details can be seen in the Supplementary Files). A preliminary analysis indicated that factor analysis was appropriate (Cronbach’s α = 0.829 and KMO = 0.83). Therefore, through exploratory factor analysis, we finally extracted a factor and named it the perceived government coping validity based on the criterion of an eigenvalue greater than 1.

##### Control Variables

Following previous studies [48,72], the control variables used in this paper included gender, age, academic qualifications (transformed into years of education in the model), census register, and whether the respondents are returnees (see Table S3 for details).

### 2.3. Modeling

In this study, we conducted a descriptive statistical analysis and regression analysis. First of all, we conducted a cross-tabulation description on nominal variables such as different genders and regions, different risk perceptions, and individuals’ responding behaviors, including stopping work or employment, suspending entertainment and travel, and stockpiling of goods. At the same time, we also described and analyzed the correlation between individual risk perception consequences, perceived government coping validity, and individual responding behaviors. Secondly, we set up three different logit models to explore how the core independent variables such as risk perception and perceived government coping validity affected individual responding behavior. All the models built were through the F-test, and the coefficients in the models were calculated through the *t*-test, with the significance marked by an asterisk.

Since the explanatory variables in this paper were ordinal measured variables, the independent variables were gradually included in the model to explore the links between the independent variables and several different dependent variables. We therefore used the generalized ordered logistic regression model described by Williams [76] with the following model setup:

$$\mathrm{Pr}(Y_{I}>j)=\frac{exp\left(\alpha_{j}+X_{i}\beta_{j}\right)}{1-\left[exp\left(\alpha_{j}+X_{i}\beta_{j}\right)\right]}\;,j=1,\;2,\;3,\;4,\;5$$

In this model, we set “*j*” to transform the multi-classification problem into a classification problem with classification objectives of {1…*j*} and {*j* + 1…*k*}. The logit defined based on these two classes represented the logarithm of the cumulative probability of belonging to the *k* − *j* classes concerning the cumulative probability of the previous “*j*” classes, called the cumulative dominance model. Therefore, in this analysis, the dependent variable *Y* took values from 1 to 5, so the model was shown as follows:

$$P_{1}=P\left(y=1|x\right)=\frac{exp\left(\alpha_{1}+\beta_{x}\right)}{1+exp\left(\alpha_{1}+\beta_{x}\right)}\;p_{1}=p\left(y=1\right)=p_{1}$$

$$P_{2}=P\left(y=2|x\right)=\frac{exp\left(\alpha_{2}+\beta_{x}\right)}{1+exp\left(\alpha_{2}+\beta_{x}\right)}\;\;p_{2}=p\left(y=2\right)=p_{2}-p_{1}$$

⋯

$$P_{5}=P\left(y\leq 5|x\right)=1\;\;\;p_{5}=p\left(y=5\right)=1-p_{4}$$

The first model represented the relationship between the probability P of the dependent variable y taking the first value and x, and the second model represented the relationship between the cumulative probability P of y taking the first two values and x. These models had different constant terms and identical regression coefficients. $p\left(1\right)=p_{1}$ for the probability of y taking the first value, $p\left(2\right)=p_{2}-p_{1}$ for the probability of y taking the second value, and $p\left(5\right)=1-p_{4}$ for the probability of y taking the fifth value. The analyses in this paper were based on Stata 16.0.

## 3. Results

### 3.1. Descriptive Analysis

According to Table 1, in terms of demographic background variables, among the 1537 people who took this survey, males accounted for 31% and females accounted for 69%. The chi2 test showed there was a significant difference between their suspension of entertainment and travel, stopping work or employment, and stockpiling of supplies. The age range was between 6 and 66 years old, with a mean age of 26 years old, and the proportion of people who returned to their hometown was 45.7%. The proportion of rural residents was 42.84%, and the proportion of urban residents was 57.16%. The chi2 test indicated that individuals living in towns versus rural areas reported significantly higher tendencies to stop working and higher stockpiling behavior, just as there were substantial differences in the suspension of entertainment and the stockpiling of supplies between those who returned home versus those that did not.

Further, in terms of people’s perceptions of the COVID-19 pandemic, 94.9% of the people said they “agree” that it was “highly contagious”, 93.8% of the people were afraid of the virus, and 54.4% of the people agreed that “the virus would get out of control”. Further analysis showed some significant differences between those who agreed with these views and those who did not.

The correlation analysis showed that people’s opinions about the COVID-19 pandemic, namely that it was “highly contagious” and “the virus will get out of control”, significantly correlated with their suspension of entertainment and travel, stopping work or employment, and stockpiling of supplies (*p* < 0.05).

Moreover, in Table 2, the correlation analysis showed that in terms of people’s perception of the consequences of the COVID-19 outbreak, the perception of “anxiousness” of the consequences was strongly correlated with people’s response to the pandemic in terms of entertainment and travel, stopping work or employment, and stockpiling supplies (*p* < 0.01). Simultaneously, there was a strong correlation between the potential impacts of the virus on people’s response to entertainment and travel and the stockpiling of supplies (*p* < 0.05), while there was no correlation with response to stopping work or employment (*p* > 0.05).

Perceived virus “controllability” was strongly correlated with people’s tendency to stop work or employment as well as stockpile supplies during the outbreak (*p* < 0.05) but was not significantly correlated with people’s entertainment and travel behaviors (*p* > 0.05). Finally, in terms of the people’s perception of the government’s coping validity in the early stages of the pandemic, it was strongly correlated (*p* < 0.05) with their response to the pandemic in terms of entertainment and travel, stopping work or employment, and stockpiling supplies.

### 3.2. Regression Results

To explore the relationship between risk perception, perceived government coping validity, and individual responding behaviors, three models were separately developed for each of the three measures of people’s behavior in response to the pandemic: entertainment and travel, stopping work or employment, and stockpiling supplies.

As seen in Model 1 (Table 3), regarding the control variables, only gender and returning home significantly affected people’s suspension of entertainment and travel, while age, academic qualifications, and rural or urban registry did not. In terms of people’s perceptions of the COVID-19 pandemic, the more strongly people agreed that the COVID-19 pandemic was “highly contagious”, the more likely they were to suspend entertainment and travel. The effect of people agreeing that the virus was “highly contagious” on their likelihood to suspend entertainment and travel was increased by 1.1 times (e^0.747^ − 1) compared to those who disagreed. Furthermore, in terms of anxiousness and potential impacts of the consequences of the COVID-19 pandemic, each unit increase in both perceptions increased the probability of suspending entertainment and travel by 72.8% (e^0.547^ − 1) and 45.4% (e^0.374^ − 1), respectively, which showed that anxiousness and perception of potential impact had significant positive effects on people’s suspension of entertainment and travel. At the same time, people’s fear of the virus and their risk perceptions on the dimensions of the virus potentially getting out of control did not lead people to suspend entertainment and travel (*p* > 0.05). Moreover, the controllability of the consequences of the pandemic among people did not have a significant effect on people’s suspension of entertainment and travel either (*p* > 0.05). This showed that Hypothesis 1 was partially verified.

In terms of perceived government coping validity, people’s positive evaluation of the perceived government coping validity during the pandemic significantly negatively affected people’s suspension of entertainment and travel. The more effectively the government coped with the pandemic, the lower the probability of people’s suspension of entertainment and travel was (1 − e^−0.157^, *p* < 0.05), and the more people were able to boldly and safely carry out their entertainment and travel. Thus, Hypothesis 2 was verified.

From the results of Model 2, regarding the control variables, gender, academic qualifications, and area rather than age or returning home were associated with stopping work or employment. As for the core independent variables, the more “contagious” people perceived COVID-19 to be, the more likely they were to stop work or employment (*p* < 0.05), and people’s perception that “the virus would get out of control” would have a significant positive effect on people “stopping working or employment”. The stronger this perception was, the more likely people were to stop work or employment (e^0.472^, *p* < 0.001). In addition, the higher people’s perceived “anxiousness” of the consequences of the COVID-19 pandemic was, the greater the probability was of them stopping work or employment. That is, each unit increase in people’s “anxiousness” about the consequences of the pandemic led to a 45.4% (e^0.347^ − 1) increase in the probability of people stopping work or employment (*p* < 0.001). In contrast, for every one-unit increase in the perceived “potential impacts” of the consequences of the COVID-19 pandemic, the probability of stopping working or employment increased by 11.9% (e^0.113^ − 1), which showed that the perception of potential impacts harmed people’s work or employment.

Meanwhile, there was no significant effect of “fear of virus” on people stopping work or employment. Hypothesis 3 was thus partially verified. Finally, the probability of people stopping work or employment decreased by 14.5% (1 − e^−0.157^) for each unit increase in perceived government coping validity, which meant that the perceived government coping validity during the pandemic had a significant adverse effect on people stopping work or employment.

From the result of Model 3, among the control variables, only gender, academic qualifications, and area affected people’s stockpiling of supplies, while age and returning home did not. As for the core independent variables, people’s perception of the “contagion” of COVID-19 and their fear of the virus did not affect their behavior of stockpiling supplies. However, people’s perception of the virus getting out of control significantly affected the probability of stockpiling supplies (e^0.366^ − 1, *p* < 0.001). Simultaneously, people’s anxiousness about the social consequences of the COVID-19 pandemic and their perception of the potential impacts had significant impacts on the stockpiling of supplies. When these two kinds of consequences were perceived to increase by one unit, the probability of stockpiling supplies increased by 70.1% (e^0.531^ − 1) and 22.1% (e^0.199^ − 1), respectively. However, the perception of controllability did not affect the stockpiling of supplies (*p* > 0.05), and Hypothesis 4 was thus partially verified.

For perceived government coping validity, the regression coefficient was −0.232 (*p* < 0.05). This meant that for each unit increase in people’s perception of perceived government coping validity, the probability of people’s stockpiling decreased by 20.7% (1 − e^−0.232^), which showed a significant negative effect of perceived government coping validity on the stockpiling of supplies—i.e., the more effective the government was in responding to the pandemic, the less likely people were to stockpile. Thus, Hypothesis 5 was confirmed.

## 4. Discussion

This study examined the relationship between individual risk perceptions, the government’s coping validity, and the three responding behaviors that people engaged in regarding the suspension of entertainment and travel, stopping work or employment, and stockpiling supplies through the analysis of data from an online survey conducted during the initial phase of the COVID-19 pandemic.

It was found that both perceptions of COVID-19 itself and perceptions of the consequences of COVID-19 had significant effects on coping behaviors such as the suspension of entertainment and travel, stopping work or employment, and stockpiling supplies. However, the specific dimensions of perceptions of COVID-19 itself and its consequences differed somewhat for these three coping behaviors.

### 4.1. The Relationship between Risk Perception and Stopping Work or Employment

First, it was interesting to conclude that the perception of contagiousness and fear did not cause people to stop working or employment. However, the perception of the uncontrolled nature of COVID-19 caused people to stop working or employment. Previous studies revealed that the high rate and uncontrolled nature of COVID-19 infection brought about different levels of unemployment in various countries, with people losing their income and even their source of livelihood [77]. For example, the most stringent city closure measures taken by the Indian government at the beginning of the pandemic caused 1.3 billion inhabitants to suspend their social and economic activities, with a 57% drop in income and a 73% reduction in working hours [78]. As can be seen, the impact of COVID-19 left people in a precarious state of employment, causing millions of people to lose their jobs [79,80].

People were faced with the dilemma of continuing to work at the risk of being infected with COVID-19 or not working, which significantly reduced the chances of being infected but could lead to unemployment. Once unemployed, financial resources for survival would be gone. Between the risk of infection and working for a living, most people usually chose to continue working. However, if COVID-19 were more than just highly contagious and out of control, then it would mean that just going out to work would undoubtedly infect people and possibly cost lives, and that was when people would stop working. Many medical professionals were still involved on the front line of the fight against COVID-19 at the risk of being infected, and most takeaway workers, food delivery workers, and transport workers continued to work in high-risk areas of the pandemic [78,81,82]. At the same time, studies showed that while stopping work or employment was not affected by people’s fear of the virus, there was a significant positive relationship between anxiety perceptions of the consequences of COVID-19 and stopping work or employment. Much of this anxiousness about the consequences was derived from social amplification and reinforcement effects, thus deepening the level of apprehension about COVID-19 [83].

### 4.2. The Relationship between Risk Perception and Suspension of Entertainment and Travel

Second, there was a significant relationship between the perception of COVID-19 itself, especially the perception of the strong contagiousness of COVID-19, and the suspension of entertainment and travel. In contrast, fear of the virus and of it being out of control did not lead to the suspension of entertainment and travel. This conclusion might seem challenging, but it has a profound logic. We believe this was mainly due to the different perceptions of the two characteristics and strong contagiousness. People were less alert to COVID-19 in its early stages. However, when people discovered its high contagiousness, risk-averse human instincts were triggered into strong risk perceptions, influencing individual behaviors in response to the risk [8,84,85,86]. The contagious cognitive characteristics were based on the accumulation of experiences that occurred in the past, while the fear of COVID-19 and the attribute that COVID-19 might get out of control in people’s cognition originated from people’s subjective judgment, which people did not tend to use as a basis for reducing trips out without the accumulation of past experiences as support. A previous study also confirmed that people’s travel—for example, to visit natural tourist attractions such as national parks—continued despite subjective recognition of specific hazards of COVID-19 [87]. However, once people realized the social harm caused by COVID-19, they would suspend entertainment and travel to prevent COVID-19 infection. As the perception of the social consequences of COVID-19 was developed after the impact on itself, the surrounding environment, and society, this perception went beyond subjective judgments to become “real”. Random travel not only increased the likelihood of acquiring COVID-19 but also expanded the means of transmission of COVID-19 and increased the negative impacts on society.

### 4.3. The Relationship between Risk Perception and Stockpiling of Supplies

Third, it was found that the fear of COVID-19 and the perception of COVID-19 being uncontrolled significantly positively affected people’s stockpiling behavior. However, the perception of the strong contagiousness of the virus did not have such a significant relationship to stockpiling behavior. Risk perception was an essential factor influencing people’s coping behavior, and when people were exposed to a sudden public health event such as the COVID-19 pandemic, the public tended to stockpile supplies out of self-protection and needs when they perceived high risks to prevent insufficient supplies from affecting their daily lives. This finding was consistent with the findings of previous studies which revealed that when people were exposed to significant emergencies, they developed high-risk perceptions and tended to adopt protective behaviors to reduce their risks guided by high-risk perceptions [88].

In addition, there was a significant positive effect of anxiousness and potential impacts caused by COVID-19 on the stockpiling of supplies. People’s stockpiling behavior was even reinforced after recognizing that COVID-19 caused anxiousness and potential effects. This was because when COVID-19 led to inevitable social consequences, it reinforced people’s perception of risk. Members of the public with higher levels of risk perception also had an increased need for supplies security and, thus, were more inclined to stockpile supplies. This finding was consistent with previous studies [89].

### 4.4. The Relationship between Perceived Government Coping Validity and Suspension of Entertainment and Travel, Stopping Work or Employment, and Stockpiling of Supplies

The study also found a strong relationship between the subjective evaluation of the government’s coping validity and individual responses. The more people believed that the government could cope, the less likely they were to stop entertainment, work, and stockpiling. Conversely, the more people perceived that the government could not manage effectively, the more likely they would be to suspend entertainment, work, and stockpiling. These findings are broadly similar to those of previous research, which revealed that the less confident people were in the perceived government coping validity, the more likely they were to engage in self-protective behaviors. A previous study also showed that the perceived government coping validity had a significant negative effect on stockpiling behavior—i.e., if the government did not cope effectively with the pandemic, people were likely to purchase household goods, even leading to a rush to buy [39].

### 4.5. Limitations

Although some expected results were obtained, this study still has some limitations due to the objective conditions. First, the data in this study were obtained through an online survey within a short period. The representativeness of this study may be somewhat limited due to the overall lack of clarity and boundaries of the online survey method itself. Second, in the sample of this study, the gender ratio was unbalanced and the interviewees were relatively highly educated, thus influencing the findings to some extent. However, these two indicators were not core explanatory variables and had a limited impact on the results. In addition, some studies showed that media messages affect people’s risk perceptions to some extent, thus impacting individual responses [43,90]. Media information is an essential variable influencing people’s risk perceptions and should be paid attention to in following studies. Finally, the population of this paper is only the domestic population two months after the early COVID-19 outbreak. This paper only focuses on the effects of risk perception and perceived government coping validity on individual behaviors in the early pandemic context. Perceptions may overtake people’s risk perceptions over time, and government coping strategies and various policies may change as the pandemic develops, requiring in-depth research.

## 5. Conclusions

In terms of understanding the epidemic itself, the risk perception of the highly contagious nature had a significant positive impact on the suspension of entertainment and travel and a negative impact on stopping work or employment. The perception that the virus would get out of control significantly stopped work employment and increased the stockpiling of supplies. The perception of anxiousness was positively associated with all three kinds of behaviors of people coping with the outbreak. Additionally, potential impacts were positively related to the suspension of entertainment and travel and the stockpiling of supplies. In contrast, perceived government coping validity had a significantly negative correlation with the suspension of entertainment and travel, stopping work or employment, and the stockpiling of supplies.

## Figures and Tables

**Table 1:** Descriptive analysis.

Variables		Suspension of Entertainment and Travel		Stopping Work or Employment		Stockpile of Supplies	
		Mean	Chi2 Test	Mean	Sig.	Mean	Chi2 Test
Gender	Male	4.14	*p* < 0.01	2.15	*p* < 0.01	2.98	*p* < 0.05
	Female	3.92		1.85		2.84	
Returning home	Yes	4.15	*p* < 0.01	1.99	*p* > 0.05	2.99	*p* < 0.01
	No	3.84		1.91		2.80	
Census register	Rural	3.98	*p* > 0.05	2.18	*p* < 0.05	2.99	*p* < 0.05
	Town	4.00		1.77		2.81	
Highly contagious	Disagree	3.59	*p* < 0.05	2.40	*p* < 0.05	2.94	*p* > 0.05
	Agree	4.01		1.92		2.88	
Fear of viruses	Disagree	3.81	*p* > 0.05	2.07	*p* > 0.05	2.62	*p* < 0.05
	Agree	4.00		1.94		2.91	
Virus out of control	Disagree	3.92	*p* < 0.05	1.74	*p* < 0.01	2.64	*p* < 0.01
	Agree	4.04		2.12		3.09	

**Table 2:** Descriptive analysis (continued).

Variables	Suspension of Entertainment and Travel		Stopping Work or Employment		Stockpiling of Supplies	
	Coef.	*t*-Test	Coef.	*t*-Test	Coef.	*t*-Test
Age	−0.057	*p* < 0.05	0.048	*p* > 0.05	−0.046	*p* > 0.05
Academic qualifications	0.0249	*p* > 0.05	−0.14	*p* < 0.05	−0.02	*p* > 0.05
Anxiousness	0.246	*p* < 0.01	0.172	*p* < 0.01	0.285	*p* < 0.01
Potential impacts	0.179	*p* < 0.01	0.013	*p* > 0.05	0.120	*p* < 0.01
Controllability	−0.003	*p* > 0.05	−0.100	*p* < 0.01	−0.052	*p* < 0.05
Perceived government coping validity	−0.123	*p* < 0.01	−0.091	*p* < 0.01	−1.88	*p* < 0.01

**Table 3:** Ordered logistic regression model of individual behavior.

Variable Name	Suspension of Entertainment and Travel Model 1	Stopping Work or Employment Model 2	Stockpiling of Supplies Model 3
Gender (male)	−0.488 ***	−0.454 ***	−0.268 ***
	[0.000]	[0.000]	[0.006]
Age	−0.004	0.016 ***	−0.006
	[0.483]	[0.004]	[0.219]
Academic qualifications	0.005	−0.105 ***	−0.024
	[0.818]	[0.000]	[0.275]
Returning home (yes)	−0.514 ***	−0.131	−0.225 **
	[0.000]	[0.229]	[0.022]
Urban (rural)	0.141	−0.684 ***	−0.223 **
	[0.152]	[0.000]	[0.019]
Highly contagious (disagree)	0.747 ***	−0.578 **	−0.228
	[0.002]	[0.019]	[0.320]
Fear of viruses (disagree)	−0.264	0.013	0.222
	[0.230]	[0.957]	[0.296]
Virus out of control (disagree)	−0.074	0.472 ***	0.366 ***
	[0.468]	[0.000]	[0.000]
Anxiousness	0.547 ***	0.347 ***	0.531 ***
	[0.000]	[0.000]	[0.000]
Potential impacts	0.374 ***	0.006	0.199 ***
	[0.000]	[0.902]	[0.000]
Controllability	0.019	−0.113 **	0.020
	[0.709]	[0.038]	[0.677]
Perceived government coping validity	−0.157 ***	−0.157 ***	−0.232 ***
	[0.004]	[0.004]	[0.000]
cut1	−3.415 ***	−1.983 ***	−2.435 ***
	[0.000]	[0.000]	[0.000]
cut2	−2.168 ***	−1.562 ***	−1.344 ***
	[0.000]	[0.001]	[0.002]
cut3	−1.389 ***	−0.383	−0.109
	[0.003]	[0.420]	[0.804]
cut4	0.280	0.738	1.467 ***
	[0.546]	[0.124]	[0.001]
N	1537	1537	1537
R^2^	0.0585	0.0537	0.0511

** *p* < 0.01, *** *p* < 0.001; *t*-test.

## Data Availability

The data presented in this study are available on request from the corresponding author.

## Supplementary Materials

The following supporting information can be downloaded at: https://www.mdpi.com/article/10.3390/ijerph20031982/s1, Table S1: Loadings of perceived factors of consequences of Covid-19 outbreak; Table S2: Government coping validity factor loadings table and gravel plot; Table S3: Variable assignment description statistics table (number, description statistics table has integrated this table); Figure S1. Eigenvalue gravel plot of government coping validity.
