# Supplementary material for: Risk Perception, Perceived Government Coping Validity, and Individual Response in the Early Stage of the COVID-19 Pandemic in China
Source: Int J Environ Res Public Health. 2023 Jan 21;20(3):1982. doi: 10.3390/ijerph20031982 (PMC9915099; doi:10.3390/ijerph20031982)
Supplement: Supplementary file 1 [file ijerph-20-01982-s001.zip › ijerph-2077600-supplementary.pdf]

Table S1 loadings of perceived factors of consequences of Covid-19 outbreak.

Variable Name	Mean	Std. Dev.	Factor 1	Factor 2	Factor 3
Covid-19 for me (totally indifferent - very worried)	4.14	0.748	0.679	0.280	0.082
The extent of the personal impact of Covid-19 on me (very little - very large)	3.74	0.923	0.627	0.217	0.135
The extent of the negative impact of Covid-19 on society (very small - very large)	4.54	0.62	0.296	0.637	0.009
Covid-19 belongs to (completely natural risk - completely man-made risk)	3.6	0.761	-0.055	0.680	0.077
Risk of Covid-19 (short term - long term)	3.38	1.024	0.161	0.560	-0.386
The degree of controllability of the consequence of Covid-19 (completely uncontrollable - completely controllable)	3.33	0.888	0.055	-0.218	0.765
Knowledge about Covid-19 (Totally unfamiliar - very familiar)	3.4	0.784	0.055	0.0305	0.695
Fear of infection or death on your own (not at all - terrified)	2.97	0.993	0.849	0.002	-0.072
Fear of infection from family and friends (not at all - terrified)	3.5	1.003	0.826	0.021	-0.065
Eigenvalue			3.012	1.274	1.132
Contribution rate %			30.15	12.74	11.32
KMO			0.775		
Alpha			0.69		

Table S2 Government coping validity factor loadings table and gravel plot.

Variable Name	Mean	Std. Dev.	Factor
Citizens' satisfaction with the series of measures taken by the government in coping with Covid-19	4.04	1.331	0.839
Citizens' approval of the central government's current actions in response to Covid-19	4.40	1.149	0.799
Citizens' approval of local government's current actions in response to Covid-19	4.09	1.374	0.797
Citizens' evaluation of the effectiveness of measures implemented by the Hubei Provincial Government to contain the spread of Covid-19	3.75	1.092	0.675
The extent to which citizens trust their local government to control local outbreaks effectively	4.60	1.115	0.738
Eigenvalue			2.98
Contribution rate %			59.55
KMO			0.827
Alpha			0.829

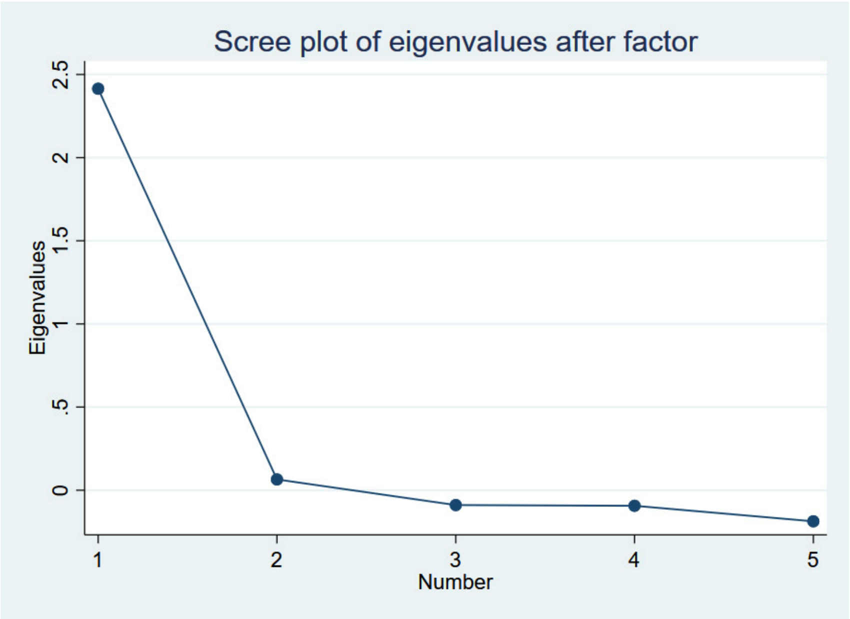

Figure S1 Eigenvalue gravel plot of government coping validity.

Table S3 Variable assignment description statistics table (number, description statistics table has integrated this table).

Variable Name		Variable assignment	Column n%
Suspension of entertainment and travel	strongly disagree	1	4.7%
	disagree	2	9.0%
	Netural	3	10.6%
	agree	4	34.3%
	strongly agree	5	41.4%
Stop work or employment	strongly disagree	1	58.2%
	disagree	2	8.9%
	Netural	3	18.7%
	agree	4	8.8%
	strongly agree	5	5.5%
Stockpile of supplies	strongly disagree	1	18.8%
	disagree	2	19.5%
	Netural	3	27.0%
	agree	4	23.6%
	strongly agree	5	11.2%

Gender	Male	1	31.0%
	Female	2	69.0%
Age	18	66	26.85
Whether to return home	Yes	0	45.7%
	No	1	54.3%
Census register	Rural	0	42.8%
	Town	1	57.2%
Highly contagious	Disagree	0	5.1%
	Agree	1	94.9%
People fear it	Disagree	0	6.2%
	Agree	1	93.8%
Out of control	Disagree	0	45.6%
	Agree	1	54.4%
